# α-Naphthoflavone Increases Lipid Accumulation in Mature Adipocytes and Enhances Adipocyte-Stimulated Endothelial Tube Formation

**DOI:** 10.3390/nu7053166

**Published:** 2015-04-30

**Authors:** Mei-Lin Wang, Shyh-Hsiang Lin, Yuan-Yu Hou, Yue-Hwa Chen

**Affiliations:** 1 School of Nutrition and Health Sciences, Taipei Medical University, 250 Wu-Hsing Street, Taipei 110, Taiwan; E-Mails: b8706083@tmu.edu.tw (M.-L.W.); lin5611@tmu.edu.tw (S.-H.L.); 2 Department of Food and Beverage Management, Mackay Medicine, Nursing and Management College, Taipei 112, Taiwan; E-Mail: s212@eip.mkc.edu.tw; 3 Cancer Research Center, Taipei Medical University Hospital, Taipei Medical University, 250 Wu-Hsing Street, Taipei 110, Taiwan

**Keywords:** α-naphthoflavone, mature adipocytes, adipogenesis, angiogenesis, aryl hydrocarbon receptor

## Abstract

The aryl hydrocarbon receptor (AhR) is a ligand-activated factor that regulates biological effects associated with obesity. The AhR agonists, such as environmental contaminants 2,3,7,8-tetrachlorodibenzo-*p*-dioxin (TCDD) and β-naphthoflavone (BNF), inhibit preadipocyte differentiation and interfere with the functions of adipose tissue, whereas the antagonist may have opposite or protective effects in obesity. This study investigated the effects of α-naphthoflavone (α-NF), an AhR antagonist, on adipogenesis- and angiogenesis-associated factors in mature adipocytes and on cross-talk of mature adipocytes with endothelial cells (ECs). Besides, the roles of the AhR on lipid accumulation and on secretion of vascular endothelial growth factor were also determined by introducing siRNA of AhR. Differentiated 3T3-L1 cells were treated with α-naphthoflavone (α-NF) (1–5 μM) for 16 h. Lipid accumulation and the expressions of AhR-associated factors in the cells were determined. The interaction between adipocytes and ECs was investigated by cultivating ECs with conditioned medium (CM) from α-NF-treated mature adipocytes, followed by the determination of endothelial tube formation. The results showed that α-NF significantly increased triglyceride (TG) accumulation in mature adipocytes, which was associated with increased expression of hormone-sensitive lipase (HSL), estrogen receptor (ER), as well as decreased expression of AhR, AhR nuclear translocator (ARNT), cytochrome P4501B1 (CYP1B1), and nuclear factor erythroid-2-related factor (NRF-2) proteins. In addition, CM stimulated formation of tube-like structures in ECs, and α-NF further enhanced such stimulation in association with modulated the secretions of various angiogenic mediators by mature adipocytes. Similarly, increased TG accumulation and vascular endothelial growth factor (VEGF) secretion were observed in AhR-knockout cells. In conclusion, α-NF increased TG accumulation in mature adipocytes and enhanced mature adipocyte-stimulated tube formation in ECs, suggesting that the AhR may suppress obesity-induced adverse effects, and α-NF abolished the protective effects of the AhR.

## 1. Introduction

Obesity is a consequence of excessive storage of lipid in white adipose tissue (WAT), which contains preadipocytes, mature adipocytes, endothelial cells (ECs), and macrophages. Correspondingly, obesity is accompanied by adipogenesis, the process that involves preadipocyte differentiation and lipid accumulation, leading to increases in adipocyte size [1,2]. Likewise, the expansion of adipose tissues is tied up with a hypoxic environment, followed by the induction of angiogenesis, *i.e*., the formation of new capillaries from existing blood vessels [3]. Various adipokines or hormones released from mature adipocytes in adipose tissues, including interleukins (ILs), leptin, vascular endothelial growth factor (VEGF), insulin-like growth factor (IGF), *etc*., were indicated to stimulate angiogenic processes [3,4,5]. Therefore, as massive amounts of lipids accumulate in WATs in obesity, intimate interactions may exist between mature adipocytes and adjacent vascular ECs, which may contribute to the physiological and pathological characteristics of adipose tissues, including the occurrence of different metabolic disorders and cancers [6,7].

Aryl hydrocarbon receptor (AhR) is a cytosolic ligand-activated transcription factor that participates in a variety of metabolic processes, including detoxification and adipogenesis. Persistent organic pollutants (POPs), such as 2,3,7,8-tetrachlorodibenzo-*p*-dioxin (TCDD), β-naphthoflavone (BNF), and polychlorinated biphenyl (PCB) are well-known environmental toxins, which bind to the AhR and exhibit different adverse biological responses. With the assistance of the aryl hydrocarbon translocator protein (ARNT), also named hypoxia-inducible factor (HIF)-1β, the activated AhR modulates expressions of various genes involved in xenobiotic-metabolizing pathways, such as cytochrome P4501A1 (CYP1A1), cytochrome P4501B1 (CYP1B1), and glutathione-S-transferases (GSTs) [8]. Both AhR and ARNT are associated with adipogenesis and angiogenesis regulation [9,10,11,12,13,14]. AhR acts as a suppressor of the adipogenic process while TCDD inhibits 3T3 preadipocytes differentiation and interferes with adipocyte functions [15,16]. WAT, especially mature adipocytes, is a reservoir and effector site for POPs due to their lipophilic and persistent properties [17,18]. The modulation of differentiation, gene expression, and functions of WAT may contribute to the adverse effects of POPs, including endocrine disrupting and, possibly, the development of obesity-associated disorders [18]. It is, then, interesting to know whether an AhR antagonist would have an opposite effects in WAT or have protective roles in development of obesity-associated disorders. α-Naphthoflavone (α-NF) is a synthetic flavonoid and a well-known AhR antagonist. Surprisingly, although α-NF has been shown to inhibit the differentiation of 3T3L-1 preadipocytes [19,20], its effects in lipid accumulation and angiogenesis on mature adipocytes, a major type of cells presenting in WAT in obese subjects, were limited. Thus, this study was designed to understand the roles of α-NF in lipid accumulation and the secretions of angiogenesis-associated factors in mature adipocytes as well as the interactions between mature adipocytes and ECs. Results obtained in this study may help elucidate roles of α-NF in adipogenesis and obesity-associated angiogenesis, and possibly, the roles of the AhR antagonists in regulating WAT functions may be clarified.

## 2. Materials and Methods

### 2.1. Chemicals

α-NF, insulin, dexamethasone, 3-isobutyl-1-methyl-xanthine, and dimethyl sulfoxide (DMSO) were purchased from Sigma Chemicals (St. Louis, MO, USA). Dulbecco’s modified Eagle’s medium (DMEM), sodium bicarbonate, fetal bovine serum (FBS), calf serum, trypan blue, and trypsin were obtained from Gibco (Grand Island, NY, USA). α-NF was dissolved in DMSO, and the concentration of DMSO in culture media was 0.1% (v/v).

### 2.2. Cell Culture

The murine 3T3-L1 preadipocyte cell line was purchased from the Bioresource Collection and Research Center (BCRC #60159; Hsinchu, Taiwan). This cell line is widely used as a model for studying adipocyte differentiation and biology [21]. Cells were grown and maintained in DMEM supplemented with 10% FBS at 37 °C in 5% CO_2_. To induce adipocyte differentiation, preadipocytes were cultivated in a differentiating medium containing insulin, dexamethasone, and 3-isobutyl-1-methyl-xanthine for 2 day and then maintained in an insulin-containing medium for another 6 day to obtain round-shaped mature adipocytes. The medium was then replaced by 1% DMEM in the presence of α-NF (1–5 μM) for 16 h, and the adipocytes and culture medium were collected for analysis.

To determine tube formation, a human endothelium-derived EA hy 926 cell line with vascular EC characteristics was used. This cell line was kindly provided by Dr. Cora-Jean Edgell (University of North Carolina, Chapel Hill, NC, USA), who established and characterized the cells [22,23], and it is now commercially available at ATCC (ATCC^®^ CRL-2922). The cells were grown as monolayers and maintained in DMEM supplemented with 10% FBS at 37 °C in 95% air and 5% CO_2_.

### 2.3. Cytotoxicity Analysis

To determine the cytotoxic effects of α-NF on mature adipocytes, cells were treated with various concentrations (1–5 μM) of α-NF for 16 h, and cytotoxicity was evaluated by the production of the formazan product, 3-(4,5-dimethylthiazol-2-yl)-5-(3-carboxymethoxyphenyl)-2-(4-sulfophenyl)-2*H*-tetrazolium (MTS) produced by live cells with a microplate reader at OD_490 nm_.

### 2.4. siRNA Transfection

To knock out the expression of the AhR in adipocytes, preadipocytes were transfected with 40 nanomolar (nM) of the stealth siRNA for AhR (siAhR) or the stealth control siRNA by the Lipofectamine 2000 transfection reagent in Opti-MEM serum-reduced medium (Invitrogen, Carlsbad, CA, USA) for 6 h. Transfected cells were then cultivated in differentiation medium to obtain mature adipocytes. Sequences for stealth siAhR and negative control siRNA were, respectively, as follows: 5′-UAACUCUGUGUUCAGCCGGUGUCUG-3′ for AhR siRNA1 and 5′-CAGAGACCGGCUGAACACAGAGUUA-3′ for AhR siRNA2.

### 2.5. Lipid Accumulation Determination

To understand the effects of α-NF on intracellular triglyceride (TG) accumulation, a marker of adipocyte differentiation and adipogenesis, mature adipocytes were stained with oil-red-O dye according to the method described by Ramírez-Zacarías *et al.* [24], and intracellular TG contents were quantified with a commercial Randox TRIGS (Cat TG213) assay kit (Randox Labs, Crumlin, UK).

### 2.6. Tube Formation Assay

To understand cross-talk between mature adipocytes and ECs, the effect of α-NF and mature adipocytes on endothelial tube formation was determined. After mature adipocytes had been treated with α-NF for 24 h, the conditioned medium (CM) was collected, centrifuged, and used for cultivating EA hy926 ECs that had been growing on BD Matrigel-coated plates for another 24 h. The ECs were next stained with calcein AM fluorescent dye (BD Biosciences, San Jose, CA, USA), and networks of vessel-like structures were observed and photographed with a fluorescent microscope.

### 2.7. Measurements of VEGF, Insulin-like growth factor 1 (IGF-1), Interleukin 6 (IL-6), and Nitric Oxide (NO)

To understand the production of angiogenic factors by mature adipocytes, concentrations of VEGF, IGF-1 and IL-6 in a culture medium of mature adipocytes were, respectively, measured with mouse DuoSet VEGF, IL-6, and IGF-1 commercial enzyme-linked immunosorbent assay (ELISA) systems (R & D Systems, Minneapolis, MN, USA). The amount of NO released from mature adipocytes was determined by reacting with the Griess reagent.

### 2.8. Western Blot Analysis

Proteins related to tube formation, adipogenesis, and xenobiotic-metabolizing pathways expressed in mature adipocytes were determined by Western blot analysis. After mature adipocytes had been treated with α-NF for 16 h, cellular proteins were extracted with lysis buffer (1% SDS and 0.15 Molar NaCl in 50 millimolar Molar (mM) Tris-HCl buffer at pH 7.5), followed by centrifugation. The extracted proteins (5 μg) were separated by 10% SDS-PAGE and then electroblotted onto a polyvinylidene difluoride membrane. Specific proteins were detected by monoclonal antibodies, including the AhR, vascular endothelial growth factor receptor (VEGFR), estrogen receptor (ER) (Santa Cruz Biotechnology, Santa Cruz, CA, USA), ARNT, CYP1B1, glycerol phosphate dehydrogenase (GPDH) (Abcam, Cambridge, MA, USA), hormone-sensitive lipase (HSL) (Origene Technologies, Rockville, MD, USA), and β-actin (Novus Bio, Littleton, CO, USA). After the addition of peroxidase-conjugated immunoglobulin G (IgG; Millipore, Billerica, MA, USA), followed by detection with Amersham enhanced chemiluminescent (ECL)™ Western Blotting Detection Reagents (GE Healthcare, Piscataway, NJ, USA). Specific visualized proteins were quantitated using Image-Pro Plus software (Media Cybernetics, Silver Spring, MD, USA).

### 2.9. Statistical analysis

Data are expressed as the mean ± standard deviation (SD). One-way analysis of variance (ANOVA) and Fisher’s least significant difference test were performed using SAS software (version 9.1, SAS Institute, Cary, NC, USA) to compare differences among groups. Differences were considered statistically significant at *p* values < 0.05.

## 3. Results

### 3.1. Effects of α-NF on Cytotoxicity and Lipid Accumulation in Mature Adipocytes

**Figure 1:**
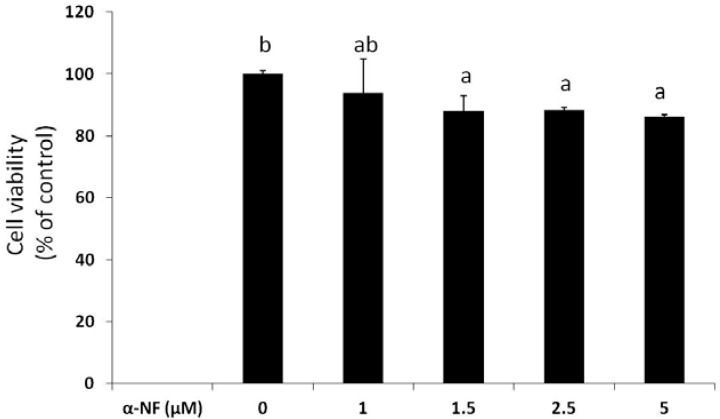
Effect of α-naphthoflavone (α-NF) on cytotoxicity in differentiated adipocytes. Cells were treated with various concentrations of α-NF for 16 h, and cell viability was measured with an 3-(4,5-dimethylthiazol-2-yl)-5-(3-carboxymethoxyphenyl)-2-(4-sulfophenyl)-2H-tetrazolium (MTS) assay kit. Values are the mean ± SD from three measurements. Data with different letters significantly differ (*p* < 0.05).

**Figure 2:**
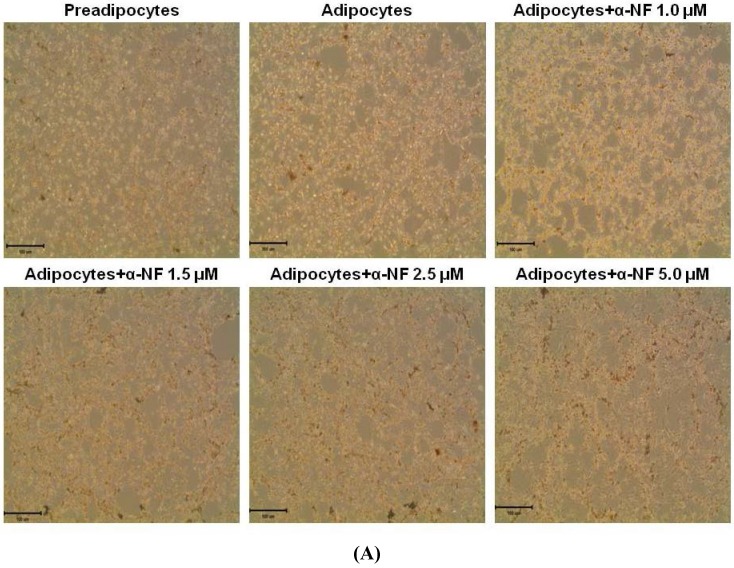

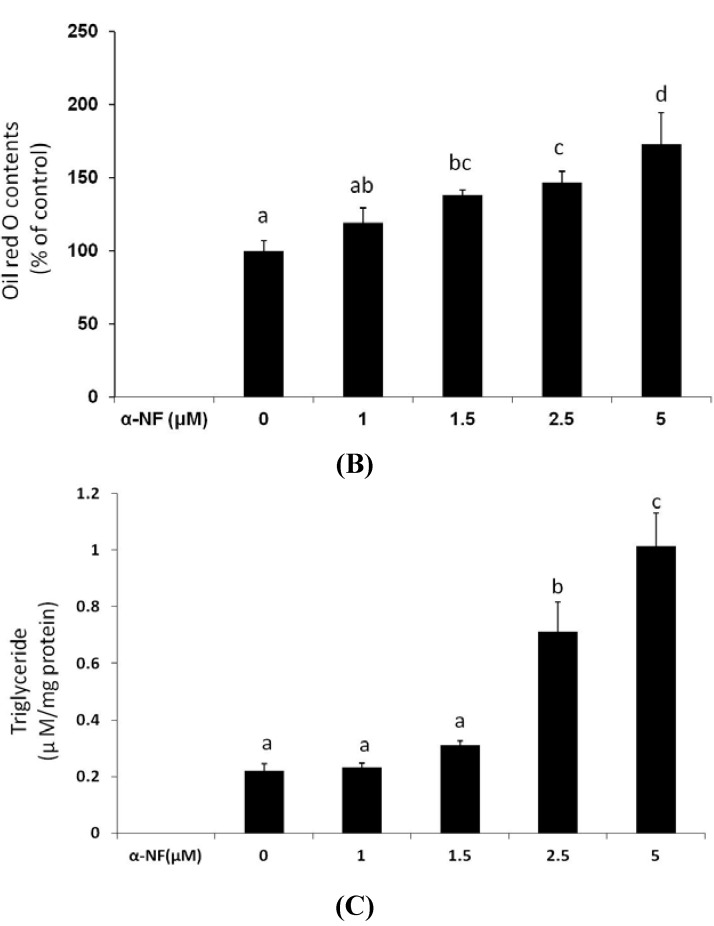
Effect of α-naphthoflavone (α-NF) on lipid accumulation in differentiated adipocytes. Cells were treated with various concentrations of α-NF for 16 h. Intracellular lipid accumulation was observed by oil-red-O staining (100× magnification) (**A**) and quantified (**B**) by the method described in “Materials and Methods”. Triglyceride (TG) contents (**C**) were quantified with a commercial kit. Pictures are representative of three independent experiments. Values are the mean ± SD from three measurements. Data with different letters significantly differ (*p* < 0.05).

Figure 1 shows that α-NF, at concentrations from 1 to 5 μM, only slightly reduced (6%–14%) the viability of differentiated mature adipocytes after 16 h of treatment. Since more than 86% of cells were viable, these concentrations were used for the following experiments. Result from Oil-red-O staining showed that more lipid droplets accumulated in mature adipocytes than in preadipocytes, which was further enhanced by α-NF treatment (Figure 2A,B). Intracellular TG contents in mature adipocytes, as expressed as μM mg^−1^ protein, was more representative to the intracellular lipid accumulation and showed a more pronounced concentration-dependent effects by α-NF treatment, ranged 105%–459% compared to those of control adipocytes (Figure 2C), whereas Oil-red-O was relative amount of OD value per plate.

### 3.2. Effects of α-NF on Adipocyte-Induced Angiogenesis in ECs

**Figure 3:**
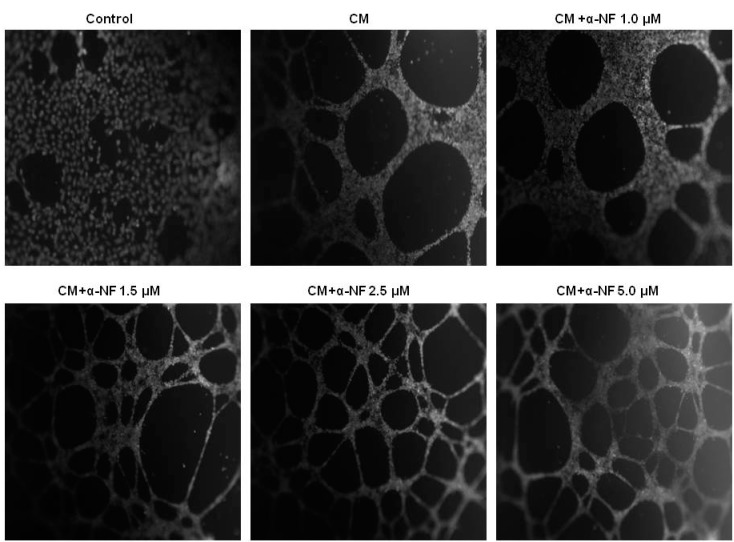
Effects of α-naphthoflavone (α-NF) on vascular tube formation in endothelial cells activated with α-NF-treated conditioned medium (CM). After full differentiation, adipocytes were incubated with Dulbecco’s modified Eagle’s medium (DMEM) containing 1% fetal bovine serum (FBS) in the presence of various concentrations of α-NF for 24 h, and CM was then used to cultivate endothelial EA hy926 cells, which were grown on Matrigel-coated plates for another 24 h. Formation of tube-like structures was observed and photographed under a phase-contrast microscope after staining with acetomethoxy derivate of calcein (calcein–AM) fluorescent dye (100× magnification). Pictures are representative of three independent experiments.

**Figure 4:**
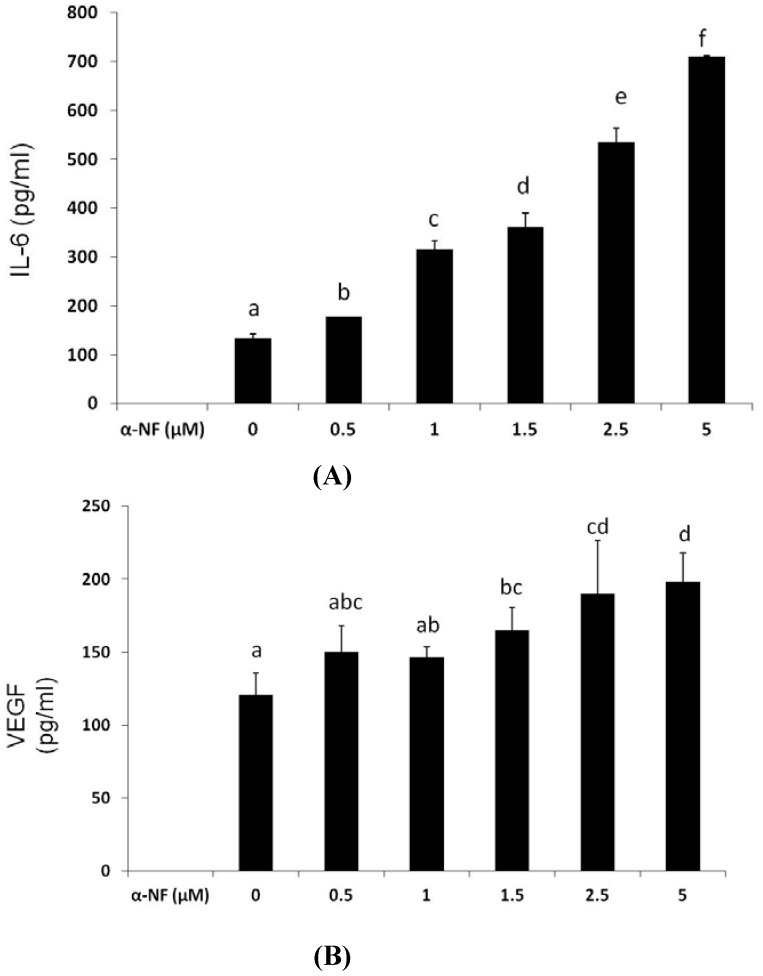

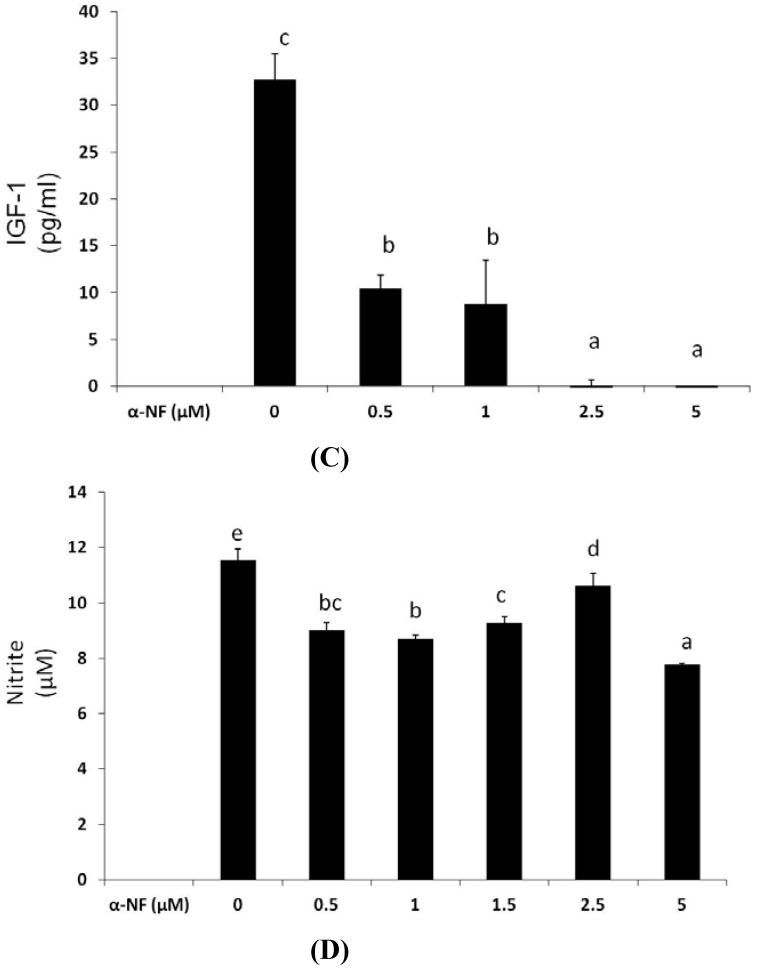
Effect of α-naphthoflavone (α-NF) on vascular endothelial growth factor (VEGF) (**A**), interleukin (IL)-6 (**B**), nitric oxide (NO) (**C**), and insulin growth factor (IGF)-1 (**D**) secretion in culture medium from differentiated adipocytes. After full differentiation, adipocytes were treated with various concentrations of α-NF in Dulbecco’s modified Eagle’s medium (DMEM) containing 1% fetal bovine serum (FBS) for 16 h. The medium was collected to analyze nitrite using the Griess reagent, IGF-1, VEGF, and IL-6 by ELISA kits. Values are the mean ± SD from three measurements, and data with different letters significantly differ (*p* < 0.05).

To understand the cross-talk between mature adipocytes and ECs, the CM collected from α-NF-treated mature adipocytes was used to cultivate EA hy926 ECs, and the formation of tube-like structures of the ECs was observed. Figure 3 shows that the CM from mature adipocytes significantly stimulated the formation of endothelial tube-like structures. Parallel to the results obtained from lipid accumulation, α-NF further enhanced interconnection networks among cells, and the enhancement was correspondingly associated with increased secretion of proangiogenic factors including VEGF (Figure 4A) and IL-6 (Figure 4B) by mature adipocytes. On the other hand, the production of NO (Figure 4C), and IGF-1 (Figure 4D), although indicated as pro-angiogenic factors, were suppressed by α-NF treatment, and IGF-1 secretion was even completely blocked by α-NF at concentrations higher than 2.5 μM.

### 3.3. Effects of α-NF on Expressions of Various Proteins by Mature Adipocytes

**Figure 5:**
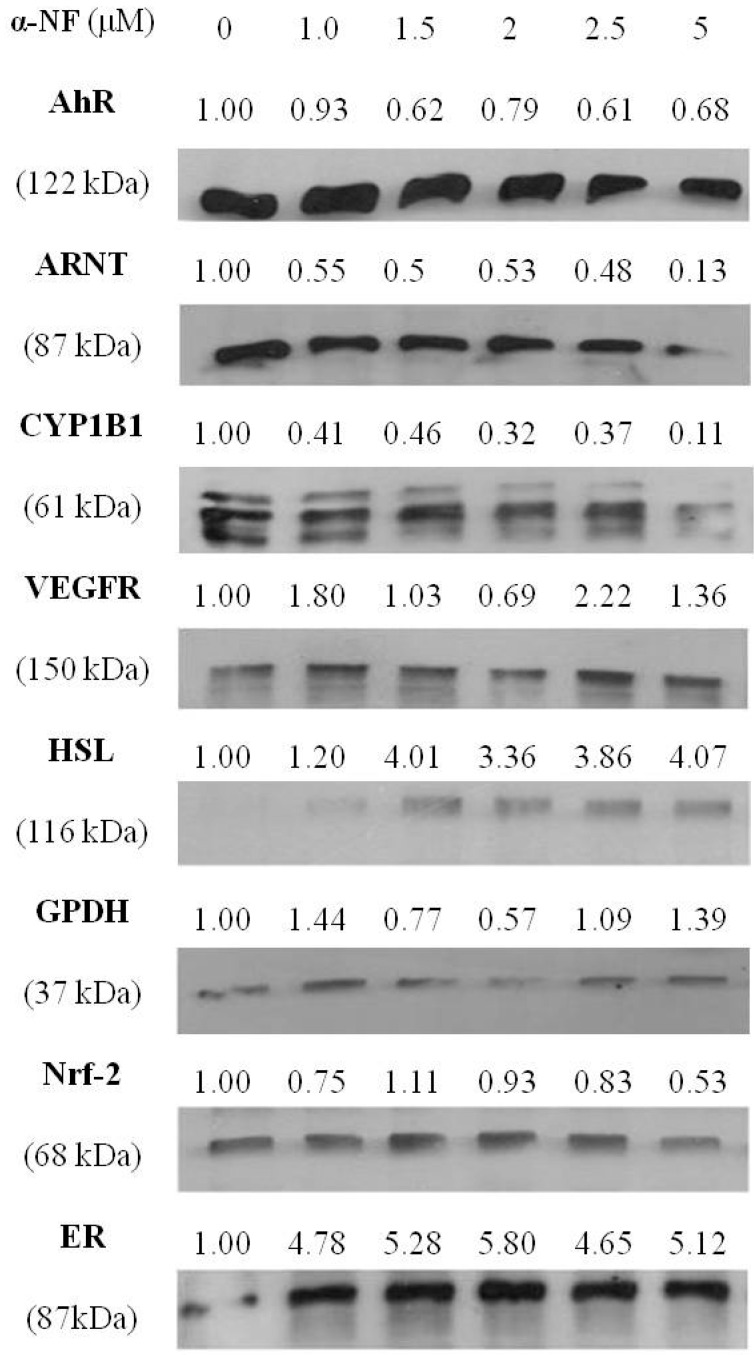
Effects of α-naphthoflavone (α-NF) on the aryl hydrocarbon receptor (AhR), the AhR nuclear translocator (ARNT), cytochrome P4501B1 (CYP1B1), estrogen receptor (ER), hormone-sensitive lipase (HSL), glycerol phosphate dehydrogenase (GPDH), nuclear factor erythroid 2-related factor 2 (NRF2), and vascular endothelial growth factor receptor (VEGFR) protein expressions by differentiated adipocytes. After full differentiation, adipocytes were treated with various concentrations of α-NF in Dulbecco’s modified Eagle’s medium (DMEM) containing 1% fetal bovine serum (FBS) for 16 h, and proteins were collected and then measured with a Western blot analysis. Levels of protein are expressed relative to the cells treated with no α-NF after being normalized by β-actin expression. Pictures are representatives of three independent experiments.

To explore the effects of α-NF on adipogenesis and angiogenesis in greater detail, expressions of associated proteins by mature adipocytes were determined. Figure 5 shows that expressions of AhR-associated proteins, including the AhR, ARNT, CYP1B1, and nuclear factor erythroid-2-related factor (NRF-2), were suppressed by treatment with α-NF. In addition, α-NF significantly enhanced HSL, an enzyme involved in TG hydrolysis, and slightly altered the expression of GPDH, a protein that participates in TG synthesis. Furthermore, the expression of angiogenic VEGFR slightly increased and ER was significantly enhanced by α-NF in mature adipocytes.

### 3.4. Effects of AhR Knockout on Lipid Accumulation and VEGF Secretion

**Figure 6:**
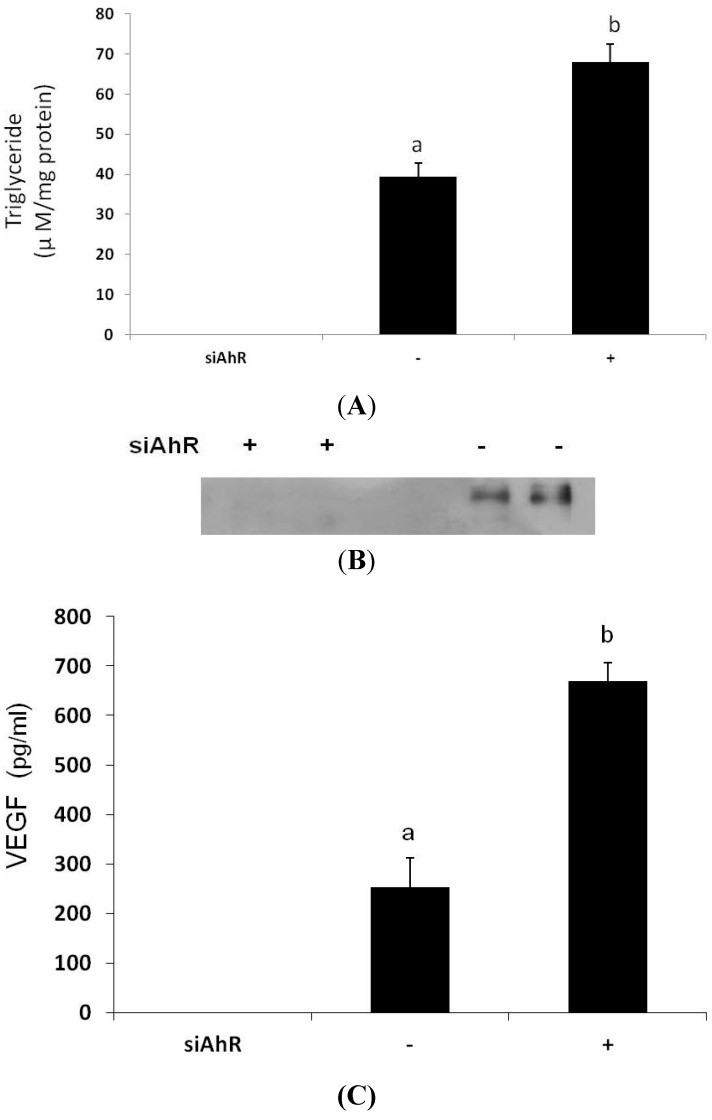
Effects of knocking out the aryl hydrocarbon receptor (AhR) on expression of the AhR (**A**), intracellular triglyceride accumulation (**B**), and vascular endothelial growth factor (VEGF) secretion by mature adipocytes. 3T3-L1 preadipocytes were transfected with siRNA for the AhR, and cells were cultivated in differentiating medium to obtain mature adipocytes. Expression of the AhR by mature adipocytes was determined by a Western blot analysis. Intracellular triglyceride (TG) contents and VEGF released into the medium were analyzed by commercial assay kits. Pictures are representative of three independent experiments. Values are the mean ± SD from three measurements. Data with different letters significantly differ (*p* < 0.05).

To clarify the roles of the AhR in α-NF-induced adipogenesis and angiogenesis, preadipocytes were transfected with siAhR followed by differentiating to mature adipocytes, and the lipid accumulation and secretion of VEGF were determined. Figure 6A shows that expression of the AhR protein was successfully blocked by siAhR transfection. Similar to the results obtained from α-NF, AhR-knockout mature adipocytes accumulated higher lipids, as determined by intracellular TG contents, than did control adipocytes (Figure 6B). Furthermore, secretion of the angiogenic mediator, VEGF, by mature adipocytes was also significantly enhanced in mature siAhR adipocytes (Figure 6C).

## 4. Discussion

Obesity is one of the major risk factors for various metabolic diseases and cancers, and the expansion of adipose tissues accompanied by enlarged adipocytes is thought to be a crucial event in these pathological conditions in which adipogenesis and angiogenesis of adipose tissues are involved. In this study, we first demonstrated that intimate cross-talk between mature adipocytes and ECs, *i.e.*, mediators released from mature adipocytes may occur through a paracrine pathway to enhance the formation of endothelial angiogenesis in WAT. In addition, both AhR antagonist α-NF and siAhR significantly increased the lipid accumulation and VEGF secretion in mature adipocytes, and α-NF further enhanced the endothelial tube formation stimulated by mature adipocytes. Correspondingly, CH223191, an AhR antagonist, has been shown to stimulate adipocyte differentiation in normal preadipcytes derived from human primary preadipcytes [25], and a natural occurring AhR agonist indole-3-carbinol possesses anti-obesity activities in high fat diet-induced obese mice [26]. These all suggest that the AhR or the AhR-mediated pathways might suppress obesity and its associated adverse effects, and α-NF abolished the protective roles of the AhR.

The AhR and ARNT are factors that mediate TCDD-, BNF-, and PCB-induced transcription of xenobiotic-metabolizing enzymes, including CYP1A, CYP1B, glutathione-S-transferase (GST), *etc.*, which participate in the metabolism of various carcinogens [8]. These lipophilic POPs are mainly accumulated and stored in mature adipocytes in WAT, and may affect WAT functions leading to the development of obesity-related disorders [17,18]. These AhR agonists have been reported to suppress preadipocyte differentiation, down regulate glucose transporting activities, modulate inflammatory responses, and interfere with estrogen functions in cultured cells and animals [11,16,17,27,28,29]. Elevated expression of CYP1A1 and CYP1B1 mRNA were also observed in adipose tissues of obese subjects [30].

Being the one of the antagonists of the AhR, which acts as a negative regulator of preadipocyte differentiation, α-NF is expected to stimulate the differentiation of the preadipocytes. Though, it was indicated that higher concentrations of α-NF (5–10 μg mL^−1^, 18.4–36.7 μM) suppressed preadipocyte differentiation [19], however, on the contrary, it was also indicated that 10 μM of α-NF showed no effect on lipid accumulation when co-administered with differentiating medium in 3T3-L1 preadipocytes [13]. Contrarily, our results showed that α-NF increased lipid accumulation in differentiated mature adipocytes, suggesting that α-NF might have more than one role in adipogenesis, in which progression of adipocyte differentiation is inhibited, whereas lipid accumulation by mature adipocytes is stimulated. Although it is well documented that AhR dimerizes with ARNT to modulate expressions of various genes, AhR may also act through one of the TCDD-independent pathways to inhibit adipocyte differentiation [13], in which α-NF mainly inhibited the mid- and late-phases of the markers in the differentiation of the 3T3-L1 preadipocytes [19]. On the other hand, the stimulatory effects of α-NF on differentiated mature adipocytes may act through antagonizing the AhR-mediated pathway, which participates lipid metabolism [31], because we observed that α-NF significantly suppressed the expression of CYP1B1, a typical AhR target protein, and because it has been shown to reduce the TCDD-stimulated gene expressions of CYP1B1 and cytokines in adipocytes [27]. This was also supported by our siAhR results. In addition to inactivating the AhR, pieces of evidence indicate that heat shock protein 90 (hsp90), a molecular chaperone associated with the AhR, plays significant roles in the AhR-mediated activation [32,33], and hsp90 inhibitors have been shown to suppress the AhR-medicated gene expression [33]. Because beta-naphthoflavone (β-NF), an AhR agonist, has impact on regulating the hsp90-AhR complex to induce CYP1A1 [32], it is plausible to hypothesize that α-NF inhibited AhR activation by interfering with the action of hsp90. Alternatively, α-NF downregulated the expression of both the AhR and ARNT proteins, and both were closely associated with the adipogenic process. Although α-NF was slightly cytotoxic at the concentrations we used, the decreased expression of AhR and ARNT might not be due to cytotoxicity, because few other proteins, such as HSL and ER, were upregulated. On the other hand, Shimba *et al.* [34] reported that the expression of the AhR decreases during differentiation and becomes almost undetectable level after full differentiation, the AhR was detectable in this study. This may explained by different AhR antibody and detection conditions used in different studies. Antisense AhR mRNA-transfected 3T3-L1 cells and adipocyte-specific ARNT-knockout mice, respectively, exhibited higher amounts of accumulated lipid droplets and less adiposity [13,14], suggesting the AhR is more dominant in the adipogenic pathway. Unexpectedly, the expression of HSL, the rate-limiting enzyme in lipolysis, was significantly stimulated by α-NF in our study. Because HSL activity was reported to mainly be regulated at the post-translational level, *i.e*., phosphorylation [35,36], the exact roles of these proteins in α-NF-induced adipogenesis require further investigation.

In addition to increasing lipid accumulation in mature adipocytes, α-NF further enhanced endothelial tube formation stimulated by CM from mature adipocytes, and such enhancement was associated with modulated secretion of various angiogenic factors by mature adipocytes, in which VEGF and IL-6 were upregulated, NO and matrix metalloproteinases (MMPs) were downregulated, and IGF-1 was completely suppressed. Mature adipocytes are able to produce and release a variety of adipokines and growth factors, and the proinflammatory mediators released from mature adipocytes may alter the cell functions in the adipose tissue. Likewise, obese subjects have higher serum levels of proinflammatory mediators, such as C-reactive protein (CRP), IL-6, and tumor necrosis factor (TNF)-α, and angiogenic factors, such as VEGF, IGF-1, MMPs, and leptin, than healthy people [37,38], and these factors are reported to participate in the pathophysiological conditions of obesity. Similar to our results, Sarkanen *et al.* [39] reported that mature human adipose tissue extract contains numerous angiogenic factors and is able to induce endothelial tube formation. Because VEGF expression is positively correlated to the size of adipocytes [40], and is enhanced by IL-6 [41], α-NF increased production of VEGF and IL-6 may be explained by the increased lipid accumulation in mature adipocytes. Although all of these mediators were indicated to be proangiogenic, these results suggest that the amounts of VEGF and IL-6 released from mature adipocytes are higher, which may act more potently on angiogenic pathways than the other cytokines. Alternatively, other adipokines secreted by mature adipocytes, such as basic fibroblast growth factor (bFGF), leptin, and adiponectin, may also contribute to the proangiogenic process [42,43]. Interestingly, α-NF, at concentrations greater than 2.5 μM, nearly completely inhibited IGF-1 secretion, although IGF-1 was indicated to be positively associated with cancers and obesity. It was shown that leptin decreases IGF-1 expression during adipocyte differentiation [44]. In addition, Zelzer *et al.* [45] suggested an intimate association between IGF-1 and the Hypoxia-inducible factor 1 (HIF-1α)/ARNT complex and modulation of hypoxia-related genes in hepatoma cells. Therefore, a complicated interrelationship among adipokines and hypoxia-induced angiogenesis may exist in adipose tissues. Nonetheless, we observed α-NF, at concentrations of 1.0–5.0 μM, further enhanced CM-induced tube formation. Because Li *et al.* [46] reported that 0.5 μM suppressed and recovered benzo[a]pyrene-inhibited tube formation in human umbilical vein endothelial cells (HUVEC), the direct effect of α-NF on the endothelial cells may exist due to the presence of α-NF in the CM of adipocytes.

In addition to ARNT, several proteins were also reported to be associated with obesity and the AhR, such as ER and NRF-2. Obesity is associated with a variety of cancers, among which a high risk of estrogen-associated breast cancer was observed in postmenopausal obese women, in which peripheral adipose tissues are the major sites for estrogen synthesis. Estrogen mainly acts through the ERs to modulate different biological responses, and intimate cross-talk between the AhR and ER signaling pathway was reported [47]. It has been indicated that the expression of aromatase, a key enzyme of estrogen synthesis, markedly increased in mature adipocytes comparing to the preadipocytes [48], and serum estrogen concentrations were positively associated with increased body mass index in postmenopausal women [49]. Because estrogen plays significant roles on regulating adipogenesis and adipokine productions in adipocytes, a positive feedback cycle may exist between estrogen secretion and ER expression in adipocytes. Likewise, NRF-2, a transcription factor involved in regulating expressions of antioxidative enzymes, may also be associated with the AhR [50]. Parallel to increasing TG accumulation, α-NF enhanced ER and suppressed NRF-2 expressions in mature adipocytes, and may thus be correlated with the increasing breast cancer risk in obese subjects. Furthermore, because activation of the AhR directly regulates NRF-2 expression followed by modulation of xenobiotic-metabolizing enzymes [51], it is plausible to hypothesize that the decreased NRF-2 by α-NF is mainly due to decreased expression of the AhR and/or ARNT protein followed by suppression of CYP1B1. These all imply that α-NF may exacerbate the development of obesity-associated disorders in obese subjects.

## 5. Conclusions

In summary, α-NF significantly enhanced lipid accumulation and increased VEGF secretion by mature adipocytes. In addition, α-NF enhanced mature adipocyte-stimulated tube formation in ECs, and such enhancement was associated with increased IL-6 and decreased IGF-1 and NO by mature adipocytes. Furthermore, α-NF down-regulated the expressions of the AhR, ARNT, CYP1B1, and NRF-2, but up-regulated the HSL and ER proteins. These suggest that the AhR may suppress obesity-induced adverse effects, and its antagonist α-NF abolished the protective effects of the AhR.
